# Development and evaluation of a pocket card to support prescribing by junior doctors in an English hospital

**DOI:** 10.1007/s11096-015-0119-y

**Published:** 2015-05-12

**Authors:** Matthew Reynolds, Elina Larsson, Richard Hewitt, Sara Garfield, Bryony Dean Franklin

**Affiliations:** Centre for Medication Safety and Service Quality, Imperial College Healthcare NHS Trust and UCL School of Pharmacy, London, UK; North West Thames Foundation School, London, UK; Department of Medicine, Imperial College London, London, UK

**Keywords:** Errors, Junior doctor, Medication, Prescribing, Reference source, Safety, United Kingdom

## Abstract

*Background* Junior doctors do most inpatient prescribing, with a relatively high error rate, and locally had reported finding prescribing very stressful. *Objective* To develop an intervention to improve Foundation Year 1 (FY1) doctors’ experience of prescribing, and evaluate their satisfaction with the intervention and perceptions of its impact. *Methods* Based on findings of a focus group and questionnaire, we developed a pocket Dose Reference Card (“Dr-Card”) for use at the point of prescribing. This summarised common drugs and dosing schedules and was distributed to all new FY1 doctors in a London teaching trust. A post-intervention questionnaire explored satisfaction and perceived impact. *Results* Focus group participants (n = 12) described feeling anxious and time pressured when prescribing; a quick reference resource for commonly prescribed drug doses was suggested. Responses to the exploratory questionnaire reinforced these findings. Following Dr-Card distribution, the post-intervention questionnaire revealed that 29/38 (76 %) doctors were still using it 2 months after distribution and 38/38 (100 %) would recommend ongoing production. *Conclusions* FY1 doctors reported feeling stressed and time pressured when prescribing; this was perceived to contribute to error. A pocket card presenting common drugs and doses was well-received, perceived to be useful, and recommended for on-going use.

**Electronic supplementary material:**

The online version of this article (doi:10.1007/s11096-015-0119-y) contains supplementary material, which is available to authorized users.

## Impact of findings on practice

- Foundation Year 1 (FY1) doctors perceived time pressure and lack of access to information to be sources of stress and to potentially contribute to erroneous prescribing, suggesting these as areas for intervention.
- A locally relevant pocket reference guide developed with input from clinical pharmacists may be useful in improving FY1s’ experience of prescribing; ours was widely used and recommended for ongoing use.

## Introduction

Prescribing errors occur in up to 15 % of medication orders in UK hospitals [1–6], comparable to international figures [7]. Interventions are therefore needed. There are many reasons why newly qualified Foundation Year 1 (FY1) doctors are a suitable target for such interventions. First, FY1 doctors do most of the prescribing in the UK hospital inpatient setting [4]. Second, while there is variation in reported junior doctors’ prescribing error rates [8], FY1 doctors are reported to have a prescribing error rate twice that of consultants [4]. Third, they are more readily accessible as a group than their senior counterparts. Fourth, good prescribing habits learnt early will hopefully be retained throughout a doctor’s career. Finally, FY1 doctors report lower confidence in their prescribing skills than FY2 doctors [9], identify that lack of knowledge of prescribing contributes to errors [10] and locally, reported finding prescribing very stressful with concerns about errors. We wanted to develop and evaluate an intervention to address these issues.

### Aims

To investigate FY1s’ views on prescribing, and develop and evaluate an intervention to improve their experience of prescribing.

## Ethical approval

This work met criteria for local audit and improvement activities exempt from NHS ethics review, and was registered locally as an audit.

## Methods

### Setting

Initial development work took place across an educational organisation that provided training to 270 FY1 doctors regionally. The intervention was then evaluated at a single London teaching hospital trust in which 82 FY1s worked across three hospital sites. In line with most UK hospitals, FY1 doctors wrote a high proportion of inpatients’ medication orders, which were handwritten onto preformatted paper drug charts.

### Developing the intervention

We first conducted a focus group with 12 FY1s to explore FY1s’ attitudes to prescribing, identify factors perceived as responsible for FY1 prescribing errors, and elicit potential solutions. The discussion was recorded, transcribed and thematically analysed.

Relevant topics identified were then explored further using an anonymous questionnaire (electronic supplementary material 1), distributed to all 270 FY1s across the region. Respondents were asked about prescribing confidence and experiences of prescribing safety. Data were analysed descriptively.

### The intervention

The focus group (Table 1) and questionnaire results suggested that an easily accessible reference source for local prescribing information would be a suitable intervention. We therefore created the Dose Reference Card (‘Dr-Card’) for commonly prescribed drugs and doses, for use at the point of prescribing when it was difficult to consult more detailed references. This was designed to be a low-cost intervention to improve FY1s’ prescribing experience and support safe prescribing.

**Table 1:** Quotes from Foundation Year 1 doctor focus group

“I think it’s difficult, because on the ward round there is that time issue, it’s so quick from patient to patient, and often like they’ll say to you prescribe this, and they’ll move on”. Quote 1	
“But also I think certain things like insulin prescribing sliding scales and warfarin regimes, things that obviously they are very important, based on each patient’s own clinical situation, there’s not really, apart from the intranet and obviously the protocols on the intranet, there’s no quick, fast sort of source that we can refer to on the ward round. So I think if there was something that was made more readily available to us”. Quote 2	
“I use this crib sheet, this was given to me by a CT1 [core trainee year 1 doctor] or something, it’s been kept up to date, but it’s just got, it’s A4 [paper size], it’s got all, all the antibiotics, with doses, IV [intravenous] and oral doses, anti-emetics, all your analgesia, laxatives, urology drugs, alcoholic drugs, chlordiazepoxide, inhalers and anxiolytics”. Quote 3	

#### Dr-Card design

**Fig. 1:**
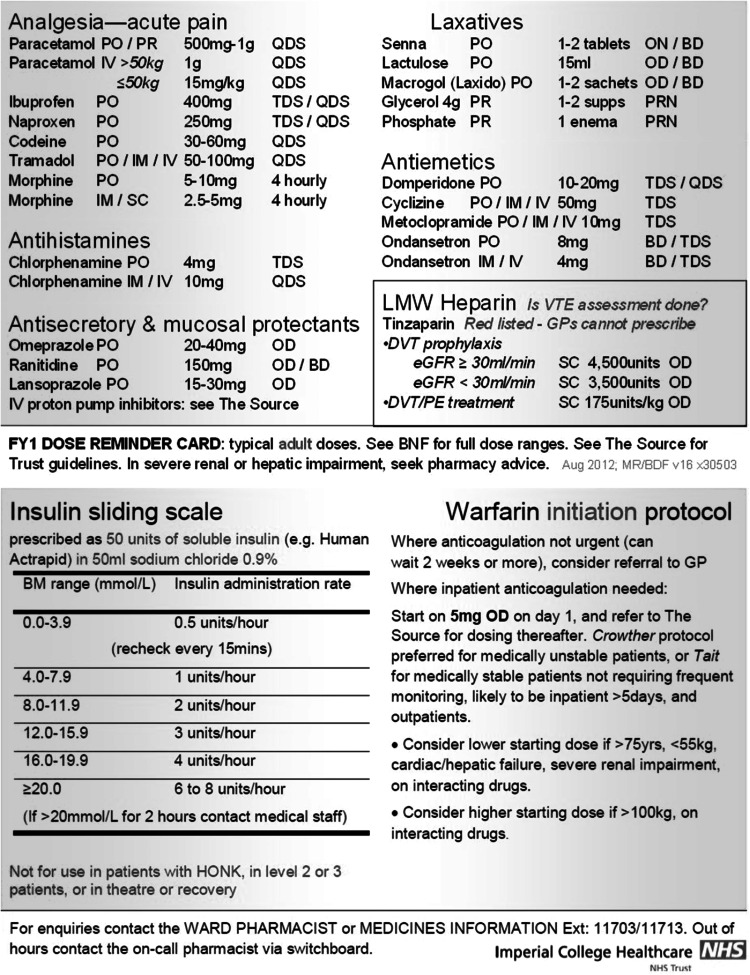
The 2 sides of the the Dr-CARD. Actual size is credit-card sized

The resulting Dr-Card (Fig. 1) included the name, dose, and route of commonly prescribed adult inpatient drugs in our local formulary. Anti-infectives were excluded as separate resources for these exist locally.

Insulin sliding scale and warfarin initiation protocols were the most frequently searched terms on the prescribing guidelines intranet site; summaries of these were also included.

The draft contents were modified in response to suggestions from specialist clinical pharmacists and FY1 representatives. The final version was approved by the Drugs and Therapeutics Committee.

The Dr-Cards (2012 version) were printed on credit-card sized laminated cardboard designed to be carried within identification badge holders or pockets; they cost £108 for 100 cards.

#### Dr-Card distribution

The Dr-Card was distributed to FY1s during teaching sessions in August 2012, the first month of the FY1 year.

#### Evaluation of the intervention

We conducted a formal post-intervention evaluation to establish the extent to which FY1s were using the Dr-Card, their views on its content, format and potential impact and to identify suggestions for improvement.

We developed a questionnaire (electronic supplementary material 2) comprising open and closed questions. A FY2 doctor and pharmacists piloted the questionnaire and confirmed its face validity.

A pre-notification email was sent to all FY1s a week before questionnaire distribution. Questionnaires were distributed at FY1 teaching sessions in October 2012, as we were interested in experiences from the first months of FY1 prescribing. Questionnaires were sent to non-attendees via internal post; data were summarised descriptively and responses expressed using the number of respondents to each question as a denominator.

## Results

### Focus group

Participants described feeling anxious and under-prepared when prescribing (Table 1) and “*daunted*” at the beginning of their FY1 year. Some expressed concern about getting “*the simple things*” wrong, both practically (e.g. missing signatures) and clinically (e.g. doses). FY1s described time pressure (quote 1) as one of the difficulties, for example not having sufficient time to look up doses to check they were prescribing correctly.

Participants identified that a quick reference ‘crib sheet’ would be a useful resource to enable them to look up commonly prescribed doses quickly (quote 2). Some had already developed their own (quote 3).

### Exploratory questionnaire

Seventy-eight (29 %) of 270 FY1 doctors from the region completed the exploratory questionnaire. One of the most frequently reported reasons for erroneous prescribing was time pressure (48/73 respondents to this question; 66 %); six (8 %) stated that lack of informative resources influenced erroneous prescribing. Other frequently chosen reasons for erroneous prescribing were lack of knowledge of the patient, and lack of pharmaceutical knowledge; these will be addressed in future interventions.

### The Dr-Card

A total of 77 (94 % of all 82 FY1s at ICHT) received the Dr-Card. Forty-three of 82 (52 %) FY1s completed the post-implementation questionnaire, of whom 41 (95 %) had received a Dr-Card and 29/38 respondents (76 %) were still using it at the time of the survey (Table 2). Four respondents who stated they “never” used it had lost their card, and one was based in paediatrics where it was not applicable.

**Table 2:** Responses to post-implementation questionnaire

	Daily	Weekly	Less than weekly	Never	Total^a^	No answer
How often did you use your Dr-Card at first?	18 (45 %)	10 (25 %)	5 (13 %)	7 (18 %)	40 (100 %)	3
How often do you use your Dr-Card now?	6 (16 %)	17 (45 %)	6 (16 %)	9 (24 %)	38 (100 %)	5

	Very satisfied	Satisfied	Neutral	Unsatisfied	Very unsatisfied	Total	No answer
How satisfied are you with the material of the Dr-Card?	21 (50 %)	16 (38 %)	5 (12 %)	0 (0 %)	0 (0 %)	42 (100 %)	1

	Plastic	Smartphone	Both	No preference	Total	No answer
What format would you prefer Dr-Card to be produced in?	8 (19 %)	4 (10 %)	28 (67 %)	2 (5 %)	42 (100 %)	1

	Yes	No	Total	No answer
Have you used the dosing guidelines for the individual drugs listed on the Dr-Card?	30 (75 %)	10 (25 %)	40 (100 %)	3
Have you used the insulin sliding scale on the Dr-Card?	17 (41 %)	24 (59 %)	41 (100 %)	2
Have you used the warfarin initiation protocol on the Dr-Card?	12 (30 %)	28 (70 %)	40 (100 %)	3
Will the Dr-Card improve patient safety?	20 (91 %)	2 (9 %)	22 (100 %)	21
Should the Dr-Card be produced next year?	38 (100 %)	0 (0 %)	38 (100 %)	5

^a^Percentages are calculated using the number of responses to that individual question as the denominator

Thirty-seven (88 %) of 42 respondents reported being satisfied or very satisfied with the material, colour and size of the card; none were dissatisfied. The majority of respondents (28/42; 67 %) wanted the card to be available as both a plastic card and smartphone application.

Most (30/40; 75 %) respondents reported using the individual drugs’ dosing guidelines; 17/41 (41 %) reported having used the insulin sliding scale and 12/40 (30 %) the warfarin initiation protocol. One respondent reported using the intranet guidelines instead.

Twenty (91 %) of 22 respondents thought the Dr-Card would improve patient safety, two (9 %) disagreed. Reasons given for improving safety included correct dosing, quicker access to medication for patients and reduced stress levels. One respondent stated that the British National Formulary (BNF) was available and would be used instead, and another that the Dr-Card would only improve safety if it was kept up-to-date and used alongside the BNF.

Finally, 38 (100 %) of 38 respondents thought the Dr-Card should be produced next year; one added that it was particularly helpful in clinical areas where common drugs had to be prescribed quickly. Some suggested additional drugs and/or protocols for inclusion.

## Discussion

A focus group and exploratory questionnaire suggested that a resource for use under time pressure may be helpful to FY1 doctors when prescribing. As some FY1s had already created their own reference sources, we formalised a novel, quick, low-cost ‘Dr-Card’, in line with local guidelines, which was distributed to all FY1s in one trust. Although dosing information and clinical guidelines are available on the trust’s intranet, doctors have intermittent access to a computer when prescribing: the Dr-Card therefore provides an accessible summary. Its portability and local relevance also complements the use of textbooks and tablet computers.

A post-implementation questionnaire revealed the majority of FY1 doctors viewed the Dr-Card positively, felt it improved safety and were still using it 2 months after distribution. All respondents thought production of the Dr-Card should continue. Patterns of use suggest the Dr-Card was used less over time, perhaps as FY1s gain experience.

### Strengths and limitations

Strengths of our study are that we developed a low-cost, practical intervention and evaluated its acceptability and perceived impact. We recognise that our intervention was based on focus group and questionnaire data from a relatively small cohort, that the questionnaire had only a 52 % response rate and that we do not have comparative demographic data for respondents and non-respondents. The evaluation was limited as we were not funded to evaluate the effect of the Dr-Card on prescribing errors.

### On-going work

Based on the evaluation, we produced an updated Dr-Card for future intakes of FY1s, presented as a more robust plastic card and also incorporated the information into a local smartphone application. Drugs and doses are reviewed annually by clinical pharmacists, FY1s and senior doctors, to ensure they remain aligned with local practice and relevant guidelines. Future research should explore the effect of quick reference guides such as the Dr-Card on prescribing errors.

## Conclusions

FY1 doctors reported feeling stressed and time pressured when prescribing; this was perceived to contribute to error. We developed the Dr-Card as a low cost intervention to provide key information at the point of prescribing. The majority of FY1 doctors carried, used, and liked the Dr-Card and thought it should be produced again. The Dr-Card is now embedded into local practice.

## Electronic supplementary material

Supplementary material 1 (DOCX 262 kb)

Supplementary material 2 (DOCX 299 kb)
