# Supplementary material for: Development and evaluation of a pocket card to support prescribing by junior doctors in an English hospital
Source: Int J Clin Pharm. 2015 May 12;37(5):762–6. doi: 10.1007/s11096-015-0119-y (PMC4594081; doi:10.1007/s11096-015-0119-y)
Supplement: Supplementary file 1 — Supplementary material 1 (DOCX 262 kb) [file 11096_2015_119_MOESM1_ESM.docx]

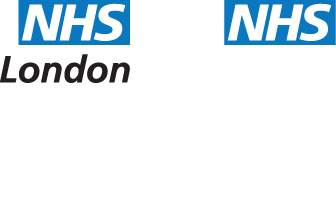

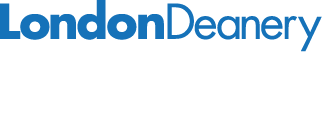


**Foundation Year 1 Prescribing Questionnaire**

***Aim:***

This questionnaire has been developed as part of a Quality Improvement Project for the North West Thames Foundation School in partnership with Imperial College Healthcare NHS Trust’s Pharmacy Department. **It explores the views of Foundation Year 1 (FY1) Doctors on the question of whether their undergraduate and postgraduate education has been sufficient in preparing them for safe prescribing on the wards.** The results will be used to help decide if education and preparation for prescribing in FY1 can be improved at both an undergraduate and postgraduate level. Participation is completely *voluntary*, and all information will be treated *confidentially*. There are **23 questions** in total.

***Questions 1-2: Demographics***

*(Please circle relevant response)*

**1. Age:**

26 or under 27-29 Over 30

**2. Gender:**

Male Female

***Questions 3-7: Pre-qualification tuition***

*(Please circle relevant response)*

**3. Where was your medical degree completed?**

Imperial College Other London Outside London Abroad

**4. Which of the following best describes the way your medical degree was taught?**

Problem-based learning Systems- based/ Integrated Traditional/ lecture- based

**5. Were you taught the practical aspects of prescribing during your medical degree, e.g. writing on a drug chart, calculating the correct drug dosage?**

Yes No

**6. Please indicate which prescribing exam(s), if any, you undertook before starting as an FY1?**

*(Please tick relevant)*

a written prescribing examination

an Objective Structured Clinical Examination (OSCE) in prescribing

other practical prescribing examination

none

**7. During my undergraduate training I received sufficient tuition to allow me to prescribe safely as an FY1 doctor**

*(Place ‘X’ in the relevant circle to indicate your level of agreement****)***

Comments……………………………………………………………………………………………….……………………………………………………………………………………………………………………………

**Strongly Disagree**

**Disagree**

**Neutral**

**Agree**

**Strongly Agree**


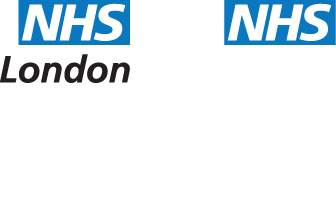

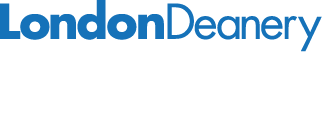


***Questions 8-11: Prescribing. Please state your level of agreement***

**8. I felt anxious about prescribing at the beginning of my FY1 year**

*(Place ‘X’ in the relevant circle to indicate your level of agreement****)***

**Strongly Disagree**

**Disagree**

**Neutral**

**Agree**

**Strongly Agree**

**8. (a) If you agreed please rank top 3 most applicable reasons (1=most applicable)**

Fear of looking incompetent

Concerns about practical aspects such as calculating drug dosages

Concerns about lack of knowledge including correct drug selection, side effects etc.

Unfamiliarity with hospital drug charts

Concern about prescribing errors and implications to patient safety

Other (please state) ……………………………………………………………………………

**9. Please indicate where you had your pharmacy induction:**

*(Please tick relevant)*

St. Mary’s Hospital West Middlesex Hospital

Hammersmith Hospital Central Middlesex Hospital

Charing Cross Hospital Chelsea and Westminster Hospital

Northwick Park Hospital I did not have a pharmacy induction

Hillingdon Hospital I do not remember my pharmacy induction

Ealing Hospital

**10. The FY1 induction prepared me adequately for prescribing on the wards**

*(Place ‘X’ in the relevant circle to indicate your level of agreement****)***

**Strongly Disagree**

**Disagree**

**Neutral**

**Agree**

**Strongly Agree**

Comments……………………………………………………………………………………………….……………………………………………………………………………………………………………………………

**11. I felt confident using the hospital drug chart when I began my FY1 job**

*(Place ‘X’ in the relevant circle to indicate your level of agreement****)***

**Strongly Disagree**

**Disagree**

**Neutral**

**Agree**

**Strongly Agree**

Comments……………………………………………………………………………………………….……………………………………………………………………………………………………………………………


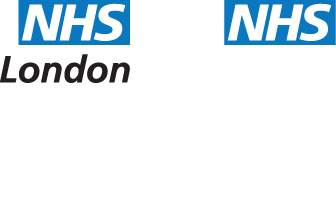

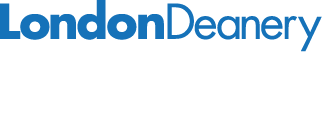


***Questions 12-18: Accurate prescribing***

**12. I feel confident prescribing in the following patient groups:**

*(Place ‘X’ in the relevant circle to indicate your level of agreement****)***

**Renal :**

**Liver :**

**Strongly Disagree**

**Disagree**

**Neutral**

**Agree**

**Strongly Agree**

**Children:**

**Strongly Disagree**

**Disagree**

**Neutral**

**Agree**

**Strongly Agree**

**Elderly:**

**Strongly Disagree**

**Disagree**

**Neutral**

**Agree**

**Strongly Agree**

**Strongly Disagree**

**Disagree**

**Neutral**

**Agree**

**Strongly Agree**

**Pregnant:**

**Strongly Disagree**

**Disagree**

**Neutral**

**Agree**

**Strongly Agree**

**13. Have you witnessed a prescribing error on the wards this year since starting as an FY1?**

*(Please circle relevant)*

Yes No

Please give a brief description of the incident:

………………………………………………………………………………………….……………………...…..………………………………………………………………………………………………………………………

**13. (a) If yes, what was the clinical consequence of this?**

*(Please tick relevant)*

no clinical consequence

patient morbidity

patient death

unknown


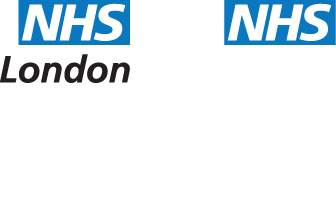

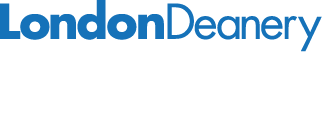

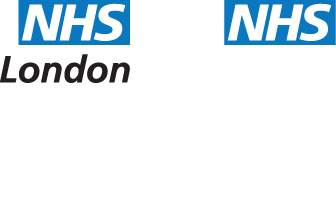

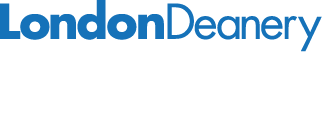


**14. I feel that I am given sufficient guidance from senior colleagues when asked to prescribe on the ward**

*(Place ‘X’ in the relevant circle to indicate your level of agreement****)***

**Strongly Disagree**

**Disagree**

**Neutral**

**Agree**

**Strongly Agree**

Comments……………………………………………………………………………………………….……………………………………………………………………………………………………………………………

**15. Which of the following do you feel has the greatest influence on risk of erroneous prescribing?**

***(Please rank top 3 in order of importance i.e. 1= most important)***

­ Time pressure

­ Lack of pharmacological knowledge e.g. drug effects, interactions, contraindications

Lack of practical prescribing skills e.g. writing on a drug chart, calculating drug dosages

­ Lack of available resources e.g. BNF, guidelines

­ Lack of available advice from senior colleagues

­ Lack of available advice from ward pharmacist

­ Pressure to prescribe unfamiliar drugs by senior colleagues

Lack of knowledge of the patient e.g. prescribing when on- call

**16. I am given adequate feedback about prescribing errors I make by senior doctors and pharmacists**

*(Place ‘X’ in the relevant circle to indicate your level of agreement****)***

**Strongly Disagree**

**Disagree**

**Neutral**

**Agree**

**Strongly Agree**

Comments……………………………………………………………………………………………….……………………………………………………………………………………………………………………………

**17. I would like more feedback on prescribing errors**

*(Place ‘X’ in the relevant circle to indicate your level of agreement****)***

**Strongly Disagree**

**Disagree**

**Neutral**

**Agree**

**Strongly Agree**

Comments……………………………………………………………………………………………….……………………………………………………………………………………………………………………………

**18. Please indicate which resource(s) you most frequently use when unsure of a drug prescription *(Please tick one or two option(s))***

BNF Pocket prescriber

The Source/ Trust protocol iPhone App

Internet resource Ward pharmacist

FY1 colleague Senior Colleague

Medicines Information

***Questions 19-23: Ongoing development***


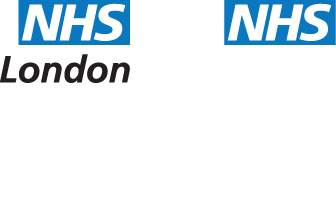

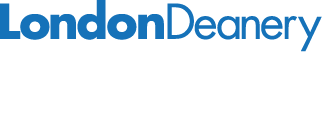


**19. I have received adequate teaching on the subject of practical prescribing during my FY1 post**

*(Place ‘X’ in the relevant circle to indicate your level of agreement****)***

**Strongly Disagree**

**Disagree**

**Neutral**

**Agree**

**Strongly Agree**

Comments……………………………………………………………………………………………….……………………………………………………………………………………………………………………………

**20. I would have liked more teaching on the subject of practical prescribing during my FY1 post**

*(Place ‘X’ in the relevant circle to indicate your level of agreement****)***

**Strongly Disagree**

**Disagree**

**Neutral**

**Agree**

**Strongly Agree**

Comments……………………………………………………………………………………………….……………………………………………………………………………………………………………………………

**21. My knowledge of clinical pharmacology, therapeutics and practical prescribing has improved since I started my FY1 post**

*(Place ‘X’ in the relevant circle to indicate your level of agreement****)***

**Strongly Disagree**

**Disagree**

**Neutral**

**Agree**

**Strongly Agree**

Comments……………………………………………………………………………………………….……………………………………………………………………………………………………………………………

**21. (a) If you agreed, please rank 3 most important reasons for this:**

*(1=most important)*

FY1 teaching on the topic

Familiarity with common drugs

Familiarity with the hospital drug charts

Support and advice from your pharmacist

Support and advice from your FY1 colleagues

Support and advice from your senior colleagues

Additional self-directed reading

Other (please state)……………………………………………………………………………

**22. I feel anxious about prescribing now**

*(Place ‘X’ in the relevant circle to indicate your level of agreement****)***

**Strongly Disagree**

**Disagree**

**Neutral**

**Agree**

**Strongly Agree**

Comments……………………………………………………………………………………………….……………………………………………………………………………………………………………………………

**23. What changes could be made to improve prescribing confidence and safety as a Foundation Year 1 Doctor,**


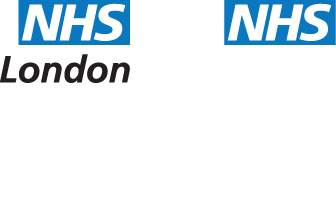

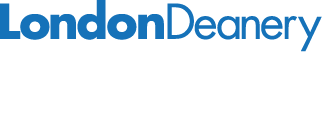


**at medical school (i.e. before qualification)?**

Comments……………………………………………………………………………………………….……...…………………………………………………………………………………………………………………………………………………………………………………………………………………………………………………………………………………………………………………………………………………………………………………………………………………………………………………………………………………………………

**during Foundation Year 1 (i.e. after qualification)?**

Comments……………………………………………………………………………………………….……...…………………………………………………………………………………………………………………………………………………………………………………………………………………………………………………………………………………………………………………………………………………………………………………………………………………………………………………………………………………………………

**Thank you for taking the time to complete this questionnaire. Your contribution is very much appreciated. Please return your completed questionnaire to a member of your Postgraduate Team.**
