# Supplementary material for: Development and evaluation of a pocket card to support prescribing by junior doctors in an English hospital
Source: Int J Clin Pharm. 2015 May 12;37(5):762–6. doi: 10.1007/s11096-015-0119-y (PMC4594081; doi:10.1007/s11096-015-0119-y)
Supplement: Supplementary file 2 — Supplementary material 2 (DOCX 299 kb) [file 11096_2015_119_MOESM2_ESM.docx]

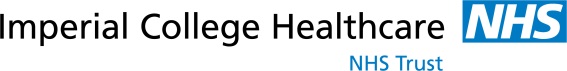

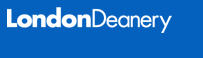


Survey of junior doctors’ views on Dr-CARDs

Following suggestions from last year’s FY1’s, we developed the Dose Reference CARD (Dr-CARD) to assist junior doctors’ prescribing. These were distributed to current FY1s this autumn. In future these will be distributed to all doctors at SMH, CXH and HH in the first week of Foundation Year 1. We would now be grateful if you could complete this short questionnaire to help us establish whether or not you found these useful, and how they could be improved. Questionnaires are anonymous and all answers are confidential.


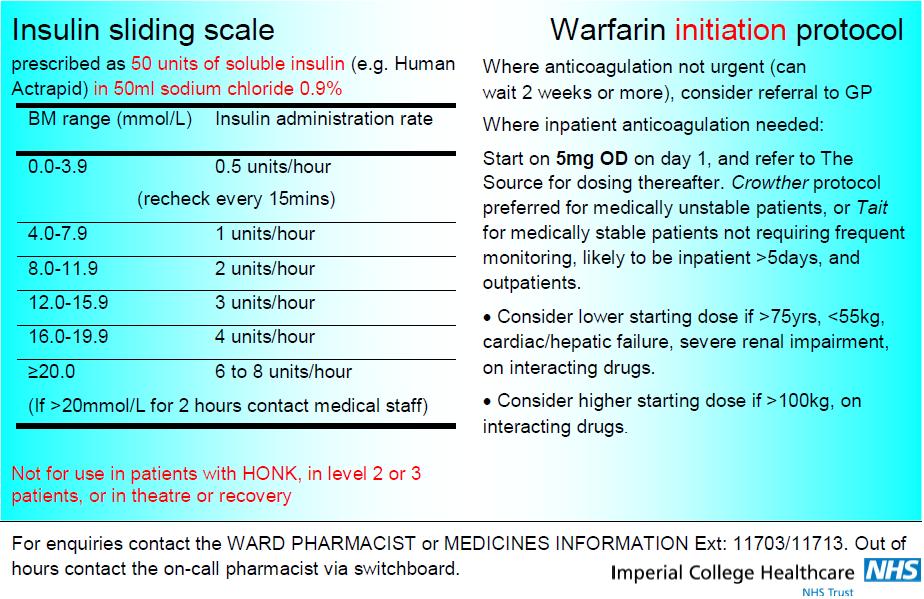

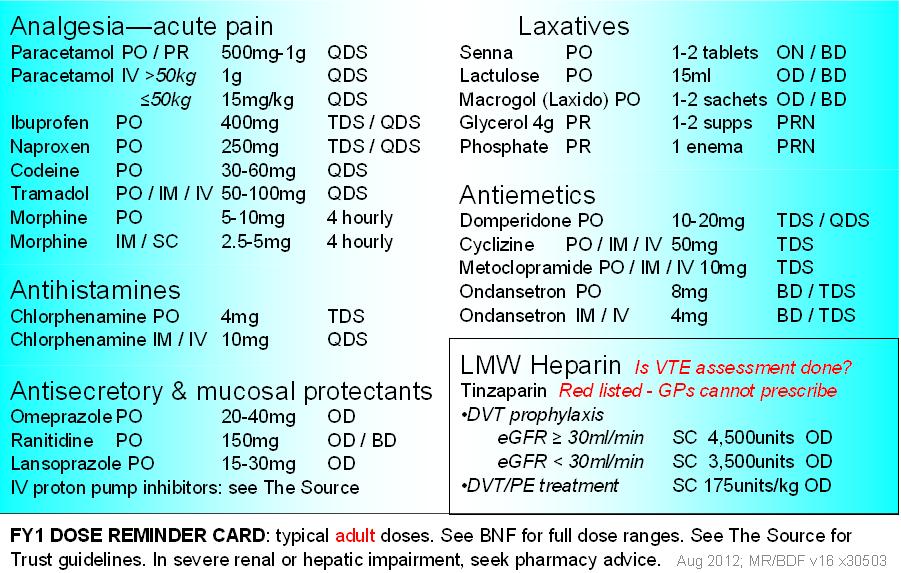


| **Section 1: Use of Dr-CARD** | | |
| --- | --- | --- |
| **1. Have you received a Dr-CARD?**  ***Please double-click and ‘check’ your response*** | Yes  No | |
| **2. If so, are you still using the Dr-CARD?** | Yes  No | |
| **3. How often did you use the Dr-CARD at first?** | Daily  Weekly  Occasionally (less often than weekly)  Never | |
| **4. How often do you use Dr-CARD now?** | Daily  Weekly  Occasionally (less often than weekly)  Never | |
| **5. How do you usually carry the Dr-CARD?** | In pocket  Threaded onto lanyard  Slotted into name badge holder  I don’t usually carry it  Other, please specify: | |
| Please continue | | |
|  | | |
| **Section 2: Format of Dr-CARD** | | |
| **6. How satisfied are you with the material (colour, size etc) of the Dr-CARD? *Please expand*** | Very satisfied  Satisfied  Neutral  Unsatisfied  Very unsatisfied | Comments: |
| **7. Would you prefer the Dr-CARD as a plastic card? *Please expand*** | Yes  No | Comments: |
| **8. Would you prefer the Dr-CARD as a smartphone app? *Please expand*** | Yes  No | Comments: |
| **9. Would you prefer the Dr-CARD both as a plastic card and a smartphone app?** | Yes  No  N/A | Comments: |
| **10. Is there any other format of the Dr-CARD that you would prefer? *Please give details.*** |  | |
|  | | |
| **Section 3: Content of Dr-CARD** | | |
| **11. Have you used the dosing guidelines for the individual drugs listed on the Dr-CARD? *Please expand*** | Yes  No | Comments: |
| **12. Have you used the insulin sliding scale on the Dr-CARD? *Please expand*** | Yes  No | Comments: |
| **13. Have you used the warfarin initiation protocol on the Dr-CARD? *Please expand*** | Yes  No | Comments: |
| **14. Is there any other key information (specific drugs or dosing protocols etc) that you think should be on the Dr-CARD? *Please give details*** |  | |
| **15. Do you think the Dr-CARD will improve patient safety? Why?** |  | |
| **16. In your opinion, should we produce cards for next year's cohort of FY1s? *Please expand*** | Yes  No | Comments: |
| **17. Do you have any further comments or suggestions?** |  | |

Thank you for your time!

Please return completed questionnaires via email by return to Matthew Reynolds, Pharmacy Department, Charing Cross Hospital. X30503. [Matthew.reynolds@imperial.nhs.uk](mailto:Matthew.reynolds@imperial.nhs.uk)
